# Associations of sickness absence and disability pension due to mental and somatic diagnoses when aged 60–64 with paid work after the standard retirement age; a prospective population-based cohort study in Sweden

**DOI:** 10.1186/s12889-021-12382-4

**Published:** 2021-12-30

**Authors:** Aleksiina Martikainen, Alice Svensson Alavi, Kristina Alexanderson, Kristin Farrants

**Affiliations:** Division of Insurance Medicine, Department of Clinical Neuroscience, Karolinska Institutet, SE-171 77 Stockholm, Sweden

**Keywords:** Cohort study, Disability pension, Extended working life, Sick leave, Mental disorders

## Abstract

**Background:**

The proportion of people working beyond age 65 is increasing. We aimed to explore whether sickness absence (SA) and disability pension (DP) due to mental, somatic, or both diagnoses when aged 60–64 were associated with being in paid work when aged 66–71.

**Methods:**

This prospective population-based cohort study included all 98,551 individuals who in 2010 turned 65 years, lived in Sweden, and were in paid work at some point when aged 60–64. Data from three nationwide registers were used with 2010 as baseline, with SA or/and DP as the exposure variables (2005–2009) and paid work as the outcome variable (2011–2016). Logistic regression was conducted to calculate odds ratios (OR) with 95% confidence intervals (CI) for the association between exposures and outcome, controlling for sociodemographic factors. The analyses were also stratified by sex.

**Results:**

Nearly half were in paid work during follow-up. Those with SA due to mental diagnoses had lower likelihood of being in paid work among both sexes (women OR: 0.76; 95% CI: 0.69–0.84; men 0.74; 0.65–0.84), while this association was smaller for SA due to somatic diagnoses (women 0.87; 0.84–0.91; men 0.92; 0.89–0.96). SA due to both mental and somatic diagnoses was associated with a lower likelihood of paid work for men (0.77; 0.65–0.91), but not women (0.98; 0.88–1.09). Regardless of diagnosis group and sex, DP had the strongest association with not being in paid work (women mental DP 0.39; 0.34–0.45; women somatic DP 0.38; 0.35–0.41; women mental and somatic DP 0.28; 0.15–0.56; men mental DP 0.36; 0.29–0.43; men somatic DP 0.35; 0.32–0.38; men mental and somatic DP 0.22; 0.10–0.51). Combined SA and/or DP demonstrated ORs in-between the diagnosis groups of SA and DP alone (e.g., mental SA and/or DP women and men combined 0.61; 0.57–0.65).

**Conclusions:**

SA and DP were negatively associated with being in paid work after the standard retirement age of 65. The association was especially strong for DP, irrespective of diagnosis group. Moreover, compared to somatic diagnoses, SA due to mental diagnoses had a stronger association with not being in paid work. More knowledge is needed on how mental SA impedes extending working life.

## Background

Raising the retirement age and getting more people in work at higher ages are concerns in many countries [1, 2]. The shift toward staying in paid work at an older age is highly needed in many Western countries due to the growing proportion of elderly [3, 4]. This is progressively possible due to increased life expectancy [5], higher number of healthy life years [6], and somewhat improved self-rated health among individuals above age 64 [7, 8].

Women tend to retire earlier than men [9] and more men than women work past age 65 [10]. This gender difference seems to become larger with age, as there are over twice as many men as women in paid work after the age of 71 [10]. Paid work past the age of 65 is also associated with factors such as country of birth [11], type of living area [12], educational level [11, 13], and family situation [12, 14–16].

In Sweden, a substantial dropout in work participation occurs at the age of 65 [17]. However, the number and proportion of people working beyond the standard retirement age of 65 has increased rapidly over the past decades [10]. The percentage of 66–71-year-olds in paid work increased from 9.8% in 1995 to 24.0% in 2010 [10]. Work participation is lower at higher ages, although a small increase has also been noted among people above age 71 [10].

## Mental health and working past age 65

To the best of our knowledge, there are no studies that have examined the association between sickness absence (SA) or disability pension (DP) due to mental diagnoses in the years leading up to the standard retirement age of 65 and being in paid work after 65. However, there are a few longitudinal studies, mostly based on self-reports, on different health conditions and paid work at an older age. In a European-wide study, 65–80-year-olds in paid work had better mental health compared to those not in paid work [18]. Moreover, not being diagnosed with a mental condition was associated with extended employment among Finnish public sector employees who had reached standard retirement age [19]. Further, people with on-going depression have been found to have a higher probability of retirement [20]. An Australian study found that poor mental health predicted overall retirement in men aged 45–75, especially early retirement [21]. However, this association was not found in women [21]. In contrast, a study from the Netherlands found no significant association between mental health and staying in paid work past the retirement age [22].

## Somatic health and working past age 65

Similar to results regarding mental disorders, there are no studies to the best of our knowledge that have investigated the association between SA and/or DP due to somatic diagnoses in the years leading up to the standard retirement age and staying in paid work after that. However, good somatic health [22] and absence of chronic disease [23] have been seen to predict working beyond the standard retirement age in the Netherlands. Indeed, physical health is shown to be better among working 65–80-year-olds compared to those not in paid work in this age group [18], a phenomenon often called the healthy worker effect. By the age of 60, age-related losses of functional capacity, such as deteriorated vision, hearing, and physical capacity, as well as non-communicable diseases (e.g., stroke, chronic respiratory disorders, and heart disease) give rise to the majority of disabilities [24]. Poor self-rated health increases the likelihood of exiting from paid work through DP before age 65 [25, 26]. In addition, a higher mean number of SA days when aged 60–64 was found in individuals exiting the workforce at the age of 65 compared to those staying in paid work past 65 [17]. A study based on survey data from 16 European countries concluded that people who still work beyond age 65 have relatively good health [18]. However, more knowledge is needed on how specific aspects of morbidity are associated with not being in paid work.

Altogether, current research shows that health is an important factor for staying in work at older age [27]. Globally, women have higher rates of morbidity [28]. In addition, SA rates are higher among women than men in many European countries [29, 30]. DP is also more common in women, at least in Nordic countries [31–33].

## Sickness absence, disability pension, and working past age 65

Some, but not most, morbidity affects people’s work capacity and can thus lead to the need for SA, or even DP if the impaired work capacity is permanent. Thus, SA and DP are not measures of morbidity per se but rather the consequences of morbidity, in terms of work incapacity. Most people with different types of diagnoses are not sickness absent due to them, as their morbidity does not affect their work capacity regarding their actual work tasks to the extent that SA/DP is needed [34]. It is, therefore, of interest to explore to what extent people are in any paid work after age 65, despite such previous (temporary or permanent) reduction in work capacity – i.e., SA or DP.

Currently, there are only a few studies on the associations of SA and DP with working past 65. Only one of these studies included information on SA diagnoses, and found that SA due to mental diagnoses was much less common after the age of 65 than in the ages 60–64, whereas the differences for somatic diagnoses were smaller [17]. Given this finding, and previous findings that show that SA spells due to mental diagnoses tend to have longer durations compared to most other SA spells [35, 36], this raises the question of whether SA or DP due to mental diagnoses could be a greater risk factor for not being in paid work after the standard retirement age than SA or DP due to somatic diagnoses. Currently, knowledge is lacking in the area.

The aim of this study was to examine the associations between having been on SA or DP due to mental, somatic, or both mental and somatic diagnoses when aged 60–64 with being in paid work at some point when aged 66–71, among women and men, adjusting for sociodemographic factors.

## Methods

### Study design

This prospective population-based cohort study examined microdata, linked at individual level, from three nationwide registers: Statistics Sweden’s LISA register (covering sociodemographic and income variables), the Social Insurance Agency’s MiDAS register (information on SA and DP, including main diagnoses according to the International Classification of Diseases (ICD-10) [37]), and the National Board of Health and Welfare’s Cause of Death register. Data for the years 2005 to 2016 were used, with 2010 as a baseline; 2005–2009 was regarded as the exposure interval and 2011–2016 as the outcome interval. The base population was all the 123,738 individuals (50.0% women) who turned 65 in 2010 and lived in Sweden in December 2010.

From this group we included everyone who had an income from work and/or received work-related benefits (SA benefits, parental leave allowance and/or work injury compensation from the National Social Insurance Agency) high enough to qualify for SA benefits in any of the five years prior to 2010 (2005–2009). In order to be eligible for SA benefits, a minimum annual work income of 24% of the price base amount (a government set amount that adjusts to annual inflation and is used to calculate different types of public benefits) was required. In order to not exclude people on long-term SA or DP, we adjusted the minimum income requirement to 75% of the minimum annual work income for SA benefits, as the SA benefit in most cases covers 75% of the lost income. This limit was an annual income of 7092 SEK in 2005 and 7704 SEK in 2009 (≈787–711 Euros/year^1^). After the applied inclusion criteria, the study cohort included 98,551 individuals (48.2% women).

### Social insurance in Sweden

In Sweden, all individuals with income from work or unemployment benefits are covered by the public sickness insurance system and can receive SA benefits if they have reduced work capacity due to disease or injury. Among employees, employers provide sick pay for the first 14 days of a SA spell, thereafter the Social Insurance Agency provides benefits. After the first seven days of a SA spell, a physician certificate, including information on diagnosis, is required. There is no time limit for the duration of SA spells, and some can last for several years. After the age of 65, some restrictions apply. Individuals aged 65–69 years can get SA benefits for up to a total of 180 days during those years, after which the Social Insurance Agency may restrict further claims if the reduced work capacity is assessed as permanent. From the age of 70, people may not claim SA benefits for more than 180 days total. All people aged 19–64 with permanently reduced work capacity due to disease or injury can be granted DP. SA benefits and DP can be granted for full- or part-time: 100, 75, 50, or 25% of ordinary work hours [38]. SA benefits cover 80% and DP about 65% of lost income, up to a certain level [39].

In Sweden, the standard retirement age was 65 years in the study period. However, the age of retirement was flexible and could start as early as 61. Most employees had the right to keep their permanent position until age 67, after which the choice to retain or terminate their employment was up to the employer. Despite the flexible pension system being designed to enable and encourage people to extend their working lives, a considerable number of people in Sweden retire before or at the age of 65 [40]. Still, many continue with some type of work.

### Exposures

The exposures were SA or DP due to mental and/or somatic diagnoses when aged 60–64 years (in the years 2005–2009). Only SA spells exceeding 14 days were included. SA and DP diagnoses were categorized in the following way:

- mental diagnosis (ICD-10: F00-F99 and Z73),
- somatic diagnosis (ICD-10: all other diagnoses, including missing diagnoses),
- both mental and somatic diagnoses,
- no SA or DP (reference category).

As SA and DP spells in many instances overlap (due to the possibility of partial SA and DP) or follow one another successively (in the working population those granted DP have first been on SA), we also conducted analyses where we used a combined variable of SA and DP. The categories were then as shown above but SA and DP were combined (SA and/or DP due to mental, somatic, or both mental and somatic diagnoses, as well as neither SA nor DP).

### Outcome

The outcome variable was whether or not an individual was in paid work at some point in 2011–2016, (i.e., when aged 66–71), defined as having an income from work or work-related benefits high enough to qualify for SA as previously described (75% of 24% of the price base amount, i.e., 7704–7974 SEK/year ≈861–873 Euros/year^2^).

### Covariates

The following sociodemographic factors were adjusted for in the analyses: sex (woman or man), country of birth (Sweden, other Nordic countries, EU-27 countries (except Denmark, Finland, and Sweden), or rest of the world (all other countries, including missing)), type of living area (large cities (Stockholm, Gothenburg, Malmö), medium-sized cities (> 90,000 inhabitants within 30 km of municipal centre), or small towns/rural areas (< 90,000 inhabitants within 30 km of municipal centre)), level of education (elementary (0–9 years, including missing), high school (10–12 years), or university/college (> 12 years)), and family situation (married or cohabiting without children under 18 years living at home, married or cohabiting with children under 18 years living at home, single without children under 18 years living at home, or single with children under 18 years living at home). Information on covariates was obtained from LISA and applies to 2010.

### Statistical analysis

Descriptive analyses were conducted. Logistic regressions were performed to calculate crude and mutually adjusted odds ratios (OR) and 95% confidence intervals (95% CI), for associations between previous SA and/or DP (when aged 60–64) in specific diagnosis groups and being in paid work when aged 66–71, crude and adjusting for sociodemographic factors. First SA and DP were considered as separate, and then as a combined variable. All analyses were conducted for all and also stratified by sex. Moreover, Nagelkerke R Squares and the Omnibus Tests of Model Coefficients were calculated. All statistical analyses were performed with IBM SPSS Statistics version 27.

## Results

Characteristics of the study cohort are presented in Table 1. About one third of all individuals had been on SA at some point when aged 60–64; 40.8% of the women and 31.3% of the men. Few individuals had been on DP (7.9%) at some point during this time period. Somatic diagnoses were the most common cause for DP (78.6% of all DP) and the clear leading cause for SA (84.6% of all SA).

**Table 1:** Characteristics of the cohort of people in Sweden in paid work when aged 60–64

	All		Women		Men	
	*N*	%	*n*	%	*n*	%
Total	98,551	100	47,461		51,090	
Sex						
Women	47,461	48.2				
Men	51,090	51.8				
Country of birth						
Sweden	90,563	91.9	43,537	91.7	47,026	92.0
Other Nordic country	4358	4.4	2430	5.1	1928	3.8
Other EU-27 country	1824	1.9	828	1.7	996	1.9
Rest of the world^a^	1806	1.8	666	1.4	1140	2.2
Type of living area						
Large city	32,020	32.5	15,850	33.4	16,170	31.7
Medium-sized city	35,116	35.6	16,907	35.6	18,209	35.6
Small city/villages	31,415	31.9	14,704	31.0	16,711	32.7
Level of education						
Elementary (≤9 years)^a^	26,838	27.2	10,991	23.2	15,847	31.0
High school (10–12 years)	42,169	42.8	20,983	44.2	21,186	41.5
University/College (≥13 years)	29,544	30.0	15,487	32.6	14,057	27.5
Family situation						
Married or cohabiting without children^b^	64,309	65.3	29,736	62.7	34,573	67.7
Married or cohabiting with children^b^	873	0.9	35	0.1	838	1.6
Single without children^b^	33,240	33.7	17,673	37.2	15,567	30.5
Single with children^b^	129	0.1	17	0.0	112	0.2
Sickness absence (SA) when aged 60–64						
No SA	63,188	64.1	28,102	59.2	35,086	68.7
Only mental SA	3258	3.3	2190	4.6	1068	2.1
Only somatic SA	29,920	30.4	15,621	32.9	14,299	28.0
Both mental and somatic SA	2185	2.2	1548	3.3	637	1.2
Disability pension (DP) when aged 60–64						
No DP	90,737	92.1	43,070	90.7	47,667	93.3
Only mental DP	1586	1.6	1037	2.2	549	1.1
Only somatic DP	6142	6.2	3301	7.0	2841	5.6
Both mental and somatic DP	86	0.1	53	0.1	33	0.1
SA and/or DP when aged 60–64						
No SA nor DP	60,497	61.4	26,600	56.0	33,897	66.3
Only mental SA and DP	3780	3.8	2526	5.3	1254	2.5
Only somatic SA and DP	31,612	32.1	16,479	34.7	15,133	29.6
Both mental and somatic SA and DP	2662	2.7	1856	3.9	806	1.6
In paid work when aged 66–71						
Yes	47,498	48.2	20,252	42.7	27,246	53.3
No	51,053	51.8	27,209	57.3	23,844	46.7

*Abbreviations*: *SA* sickness absence, *DP* disability pension

^a^ including missing

^b^ children under the age of 18 living at home

The percentage in paid work when aged 66–71 (48.2% of all in the cohort) in each sociodemographic or diagnosis group are presented in Table 2. A larger proportion of those who had not been on SA (50.6% compared to 40.5–44.3%, depending on diagnosis group), respectively not on DP (50.2% compared to 20.9–27.2%) when aged 60–64, were in paid work after age 65.

**Table 2:** Percentages in paid work when aged 66–71 among those who were in paid work when aged 60–64

	All		Women		Men	
	*n*	%	*n*	%	*n*	%
Total in paid work	47,498	48.2	20,252	42.7	27,246	53.3
Sex						
Women	20,252	42.7				
Men	27,246	53.3				
Country of birth						
Sweden	43,852	48.4	18,567	42.6	25,285	53.8
Other Nordic country	1825	41.9	980	40.3	845	43.8
Other EU-27 country	927	50.8	371	44.8	556	55.8
Rest of the world^a^	894	49.5	334	50.2	560	49.1
Type of living area						
Large city^1^	16,587	51.8	7516	47.4	9071	56.1
Medium-sized city	16,503	47.0	6911	40.9	9592	52.7
Small city/villages	14,408	45.9	5825	39.6	8583	51.4
Level of education						
Elementary (≤9 years)^a^	11,354	42.3	3746	34.1	7608	48.0
High school (10–12 years)	19,074	45.2	8237	39.3	10,837	51.2
University/College (≥13 years)	17,070	57.8	8269	53.4	8801	62.6
Family situation						
Married or cohabiting without children^b^	30,719	47.8	11,726	39.4	18,993	54.9
Married or cohabiting with children^b^	635	72.7	29	82.9	606	72.3
Single without children^b^	16,053	48.3	8482	48.0	7571	48.6
Single with children^b^	91	70.5	15	88.2	76	67.9
SA when aged 60–64						
No SA	31,986	50.6	12,555	44.7	19,431	55.4
Only mental SA	1318	40.5	849	38.8	469	43.9
Only somatic SA	13,227	44.2	6165	39.5	7062	49.4
Both mental and somatic SA	967	44.3	683	44.1	284	44.6
DP when aged 60–64						
No DP	45,530	50.2	19,235	44.7	26,295	55.2
Only mental DP	431	27.2	272	26.2	159	29.0
Only somatic DP	1519	24.7	734	22.2	785	27.6
Both mental and somatic DP	18	20.9	x	x	x	x
SA and/or DP when aged 60–64						
No SA nor DP	31,312	51.8	12,227	46.0	19,085	56.3
Only mental SA and/or DP	1473	39.0	948	37.5	525	41.9
Only somatic SA and/or DP	13,618	43.1	6317	38.3	7301	48.2
Both mental and somatic SA and/or DP	1095	41.1	760	40.9	335	41.6

*Abbreviations*: *SA* sickness absence, *DP* disability pension

x = less than 10 individuals in the category (if there are too few individuals for one sex, numbers are also withheld for the other sex in order to preserve anonymity)

^a^ including missing

^b^ children under the age of 18 living at home

Results from the logistic regression treating SA and DP as separate predictors conducted among all individuals are presented in Table 3. In this analysis, men were more likely to be in paid work compared to women. Results from the corresponding sex-stratified analyses are presented in Table 4.

**Table 3:** Odds ratios for different previous factors for being in paid work at 66–71

Variable		All individuals	
		*Crude OR [95% CI]*	*Adjusted*^*2*^ *OR [95% CI]*
Sex	Women^1^	1	1
	Men	1.54 [1.50, 1.57]***	1.55 [1.51, 1.59]***
Country of birth	Sweden^1^	1	1
	Other Nordic country	0.77 [0.72, 0.82]***	0.83 [0.78, 0.88]***
	Other EU-27 country	1.10 [1.00, 1.21]*	0.98 [0.89, 1.08]
	Rest of the world^a^	1.04 [0.95, 1.15]	0.91 [0.83, 1.00]
Type of living area	Large city^1^	1	1
	Medium-sized city	0.83 [0.80, 0.85]***	0.88 [0.85, 0.90]***
	Small town/rural area	0.79 [0.76, 0.81]***	0.87 [0.84, 0.90]***
Level of education	Elementary (≤9 years)^1,a^	1	1
	High school (10–12 years)	1.13 [1.09, 1.16]***	1.16 [1.12, 1.19]***
	University/College (≥13 years)	1.87 [1.81, 1.93]***	1.90 [1.84, 1.97]***
Family situation	Married or cohabiting without children^1,b^	1	1
	Married or cohabiting with children^b^	2.92 [2.51, 3.39]***	2.36 [2.02, 2.74]***
	Single without children^b^	1.02 [1.00, 1.05]	1.08 [1.05, 1.11]***
	Single with children < 18 years^b^	2.62 [1.79, 3.83]***	2.21 [1.50, 3.25]**
SA when aged	No SA^1^	1	1
60–64	Only mental SA	0.66 [0.62, 0.71]***	0.77 [0.72, 0.83]***
	Only somatic SA	0.77 [0.75, 0.80]***	0.90 [0.88, 0.93]***
	Both somatic and mental SA	0.77 [0.71, 0.84]***	0.95 [0.87, 1.04]
DP when aged	No DP^1^	1	1
60–64	Only mental DP	0.37 [0.33, 0.41]***	0.38 [0.34, 0.43]***
	Only somatic DP	0.33 [0.31, 0.35]***	0.36 [0.34, 0.39]***
	Both somatic and mental DP	0.26 [0.16, 0.44]***	0.26 [0.15, 0.44]***

*Abbreviations*: *SA* sickness absence, *DP* disability pension, *OR* odds ratio, *95% CI* confidence interval within 95%

^1^ reference category

^2^ mutually adjusted

^a^ including missing.

^b^ children under the age of 18 living at home.

*p < 0.05*,* **p < 0.01, ***p < 0.001

Model fit: The Omnibus Tests of Model Coefficients = 5022.598 (*p* < 0.000), Nagelkerke R Square = 0.066

**Table 4:** Odds ratios for different previous factors for paid work at 66–71; sex-stratified

Variable		Women		Men	
		*Crude OR [95% CI]*	*Adjusted*^*2*^ *OR [95% CI]*	*Crude OR [95% CI]*	*Adjusted*^*2*^ *OR [95% CI]*
Country of birth	Sweden^1^	1	1	1	1
	Other Nordic country	0.91 [0.84, 0.99]*	0.92 [0.84, 1.00]*	0.67 [0.61, 0.74]***	0.72 [0.66, 0.80]***
	Other EU-27 country	1.09 [0.95, 1.25]	0.97 [0.84, 1.11]	1.09 [0.96, 1.23]	1.00 [0.88, 1.13]
	Rest of the world^a^	1.35 [1.16, 1.58]***	1.23 [1.05, 1.45]*	0.83 [0.74, 0.93]**	0.77 [0.68, 0.86]***
Type of living area	Large city^1^	1	1	1	1
	Medium-sized city	0.77 [0.73, 0.80]***	0.85 [0.81, 0.89]***	0.87 [0.84, 0.91]***	0.91 [0.87, 0.95]***
	Small town/rural area	0.73 [0.70, 0.76]***	0.84 [0.80, 0.88]***	0.83 [0.79, 0.86]***	0.90 [0.86, 0.94]***
Level of education	Elementary (≤9 years)^1,a^	1	1	1	1
	High school (10–12 years)	1.25 [1.19, 1.31]***	1.25 [1.19, 1.31]***	1.13 [1.09, 1.18]***	1.09 [1.05, 1.14]***
	University/College (≥13 years)	2.22 [2.11, 2.33]***	2.18 [2.07, 2.29]***	1.81 [1.73, 1.90]***	1.67 [1.60, 1.76]***
Family situation	Married or cohabiting without children^1,b^	1	1	1	1
	Married or cohabiting with children^b^	7.42 [3.08, 17.89]***	9.43 [3.86, 23.05]***	2.14 [1.84, 2.50]***	2.08 [1.78, 2.43]***
	Single without children^b^	1.42 [1.37, 1.47]***	1.45 [1.39, 1.50]***	0.78 [0.75, 0.81]***	0.81 [0.77, 0.84]***
	Single with children^b^	11.52 [2.63, 50.38]**	12.77 [2.87, 56.84]**	1.73 [1.16, 2.58]**	1.68 [1.12, 2.51]*
SA when aged	No SA^1^	1	1	1	1
60–64	Only mental SA	0.78 [0.72, 0.86]***	0.76 [0.69, 0.84]***	0.63 [0.56, 0.71]***	0.74 [0.65, 0.84]***
	Only somatic SA	0.81 [0.78, 0.84]***	0.87 [0.84, 0.91]***	0.79 [0.76, 0.81]***	0.92 [0.89, 0.96]***
	Both mental and somatic SA	0.98 [0.88, 1.08]	0.98 [0.88, 1.09]	0.65 [0.55, 0.76]***	0.77 [0.65, 0.91]**
DP when aged	No DP^1^	1	1	1	1
60–64	Only mental DP	0.44 [0.38, 0.51]***	0.39 [0.34, 0.45]***	0.33 [0.28, 0.40]***	0.36 [0.29, 0.43]***
	Only somatic DP	0.35 [0.33, 0.39]***	0.38 [0.35, 0.41]***	0.31 [0.29, 0.34]***	0.35 [0.32, 0.38]***
	Both mental and somatic DP	0.33 [0.17, 0.63]**	0.28 [0.15, 0.56]***	0.22 [0.10, 0.50]***	0.22 [0.10, 0.51]***

*Abbreviations*: *SA* sickness absence, *DP* disability pension, *OR* odds ratio, *95% CI* confidence interval within 95%

^1^reference category

^2^mutually adjusted

^a^including missing

^b^children under the age of 18 living at home

*p <0.05, **p <0.01, ***p <0.001

Model fit women: The Omnibus Tests of Model Coefficients = 2499.39 (*p* <0.000), Nagelkerke R Square = 0.07

Model fit men: The Omnibus Tests of Model Coefficients = 1990.58 (*p* <0.000), Nagelkerke R Square = 0.05

### Sickness absence

Generally, having been on SA when aged 60–64 was associated with a lower likelihood of being in paid work when aged 66–71. SA due to mental diagnoses was associated with the lowest likelihood of being in paid work compared to no SA among all individuals (OR: 0.77; 95% CI: 0.72–0.83). Having been on SA due to both somatic and mental diagnoses showed no significant association with the likelihood of being in paid work past the age of 65 among all (0.95; 0.87–1.04) nor among women (0.98; 0.88–1.09), yet there was a negative association among men (0.77; 0.65–0.91).

### Disability pension

Having been on full- or part-time DP at some point when aged 60–64 was associated with a lower likelihood of working after age 65. The results from non-stratified and sex-stratified analyses were strongly in line with each other. DP due to mental diagnoses was associated with a much lower OR (0.38; 95% CI: 0.34–0.43) of being in paid work after 65 compared to those without DP. The corresponding ORs regarding DP due to somatic diagnoses were comparable. People with DP due to both mental and somatic diagnoses were the least likely to be in paid work after the age of 65, compared to those without DP.

### Sickness absence and/or disability pension

Results with the combined variable of SA and/or DP among all are shown in Table 5, and the corresponding sex-stratified analyses are shown in Table 6. The combined variable of SA and DP demonstrated ORs that largely lay in-between those of the diagnosis groups of SA and DP alone, especially for men.

**Table 5:** Odds ratios for SA and DP combined and being in paid work at 66–71

Variable		All individuals	
		*Crude OR [95% CI]*	*Adjusted*^*2*^ *OR [95% CI]*
Sex	Women^1^	1	1
	Men	1.54 [1.50, 1.57]***	1.54 [1.50, 1.58]***
Country of birth	Sweden^1^	1	1
	Other Nordic country	0.77 [0.72, 0.82]***	0.82 [0.77, 0.88]***
	Other EU-27 country	1.10 [1.00, 1.21]*	0.97 [0.88, 1.06]
	Rest of the world^a^	1.04 [0.95, 1.15]	0.88 [0.80, 0.97]*
Type of living area	Large city^1^	1	1
	Medium-sized city	0.83 [0.80, 0.85]***	0.87 [0.84, 0.90]***
	Small town/rural area	0.79 [0.76, 0.81]***	0.86 [0.83, 0.88]***
Level of education	Elementary (≤9 years)^1,a^	1	1
	High school (10–12 years)	1.13 [1.09, 1.16]***	1.16 [1.12, 1.20]***
	University/College (≥13 years)	1.87 [1.81, 1.93]***	1.91 [1.84, 1.97]***
Family situation	Married or cohabiting without children^1,b^	1	1
	Married or cohabiting with children^b^	2.92 [2.51, 3.39]***	2.37 [2.03, 2.75]***
	Single without children^b^	1.02 [1.00, 1.05]	1.08 [1.05, 1.11]***
	Single with children^b^	2.62 [1.79, 3.83]***	2.24 [1.53, 3.30]***
SA and/or DP when	No SA nor DP^1^	1	1
aged 60–64	Only mental SA and/or DP	0.60 [0.56, 0.64]***	0.61 [0.57, 0.65]***
	Only somatic SA and/or DP	0.71 [0.69, 0.73]***	0.76 [0.74, 0.78]***
	Both mental and somatic SA and/or DP	0.65 [0.60, 0.71]***	0.70 [0.64, 0.76]***

*Abbreviations*: *SA* sickness absence, *DP* disability pension, *OR* odds ratio, *95% CI* confidence interval within 95%

^1^ reference category

^2^ mutually adjusted

^a^ including missing.

^b^ children under the age of 18 living at home.

*p < 0.05, **p < 0.01, ***p < 0.001

Model fit: The Omnibus Tests of Model Coefficients = 3826.55 (*p* < 0.000), Nagelkerke R Square = 0.05

**Table 6:** Odds ratios for SA and DP combined and being in paid work at 66–71; sex-stratified

Variable		Women		Men	
		*Crude OR [95% CI]*	*Adjusted*^*2*^ *OR [95% CI]*	*Crude OR [95% CI]*	*Adjusted*^*2*^ *OR [95% CI]*
Country of birth	Sweden^1^	1	1	1	1
	Other Nordic country	0.91 [0.84, 0.99]*	0.91 [0.84, 0.99]*	0.67 [0.61, 0.74]***	0.72 [0.65, 0.79]***
	Other EU-27 country	1.09 [0.95, 1.25]	0.94 [0.82, 1.09]	1.09 [0.96, 1.23]	0.98 [0.86, 1.12]
	Rest of the world^a^	1.35 [1.16, 1.58]***	1.20 [1.03, 1.41]*	0.83 [0.74, 0.93]**	0.74 [0.66, 0.83]***
Type of living area	Large city^1^	1	1	1	1
	Medium-sized city	0.77 [0.73, 0.80]***	0.84 [0.80, 0.88]***	0.87 [0.84, 0.91]***	0.90 [0.86, 0.94]***
	Small town/rural area	0.73 [0.70, 0.76]***	0.83 [0.79, 0.87]***	0.83 [0.79, 0.86]***	0.89 [0.85, 0.93]***
Level of education	Elementary (≤9 years)^1,a^	1	1	1	1
	High school (10–12 years)	1.25 [1.19, 1.31]***	1.25 [1.19, 1.32]***	1.13 [1.09, 1.18]***	1.10 [1.05, 1.14]***
	University/College (≥13 years)	2.22 [2.11, 2.33]***	2.18 [2.07, 2.29]***	1.81 [1.73, 1.90]***	1.69 [1.61, 1.77]***
Family situation	Married or cohabiting without children^1,b^	1	1	1	1
	Married or cohabiting with children^b^	7.42 [3.08, 17.89]***	9.16 [3.78, 22.22]***	2.14 [1.84, 2.50]***	2.09 [1.79, 2.44]***
	Single without children^b^	1.42 [1.37, 1.47]***	1.44 [1.39, 1.50]***	0.78 [0.75, 0.81]***	0.80 [0.77, 0.83]***
	Single with children^b^	11.52 [2.63, 50.38]**	12.91 [2.93, 56.96]**	1.73 [1.16, 2.58]**	1.70 [1.14, 2.54]**
SA and/or DP when	No SA nor DP^1^	1	1	1	1
aged 60–64	Only mental SA and/or DP	0.71 [0.65, 0.77]***	0.61 [0.56, 0.67]***	0.56 [0.50, 0.63]***	0.55 [0.49, 0.62]***
	Only somatic SA and/or DP	0.73 [0.70, 0.76]***	0.74 [0.71, 0.77]***	0.72 [0.70, 0.75]***	0.77 [0.74, 0.80]***
	Both mental and somatic SA and/or DP	0.82 [0.74, 0.90]***	0.73 [0.66, 0.80]***	0.55 [0.48, 0.64]***	0.57 [0.50, 0.66]***

*Abbreviations*: *SA* sickness absence, *DP* disability pension, *OR* odds ratio, *95% CI* confidence interval within 95%

^1^ reference category

^2^ mutually adjusted

^a^ including missing

^b^ children under the age of 18 living at home

*p < 0.05, **p < 0.01, ***p < 0.001

Model fit women: The Omnibus Tests of Model Coefficients = 1937.54 (p < 0.000), Nagelkerke R Square = 0.05

Model fit men: The Omnibus Tests of Model Coefficients = 1367.50 (p < 0.000), Nagelkerke R Square = 0.04

## Discussion

In this prospective population-based cohort study of all 98,551 individuals in Sweden who turned 65 in 2010 and had been in paid work at some point when aged 60–64, nearly half were in paid work at some point when aged 66–71. Prior SA was negatively associated with being in paid work. However, SA demonstrated somewhat different strengths of associations depending on sex and SA diagnosis group, whereas DP had similar associations with paid work irrespective of sex and DP diagnosis group (e.g., somatic DP among all 0.36; 0.34–0.39). When combining SA and DP, the ORs largely lay in-between those of the respective diagnosis groups of SA and DP alone. To the best of our knowledge, this study is the first to explore the association between specific SA and DP diagnosis groups and being in paid work after the age of 65.

### Sickness absence

Both women and men with previous SA due to mental diagnoses were less likely to be in paid work after age 65 than those with previous SA due to somatic diagnoses, which was also found when conducting analyses on the combined variable of SA and/or DP. More knowledge is warranted on possible reasons for this result, and whether this differs by type of occupation or type of mental diagnosis. It might be that the current stigma of mental disorders [41] decreases the likelihood of being in paid work, e.g., through hesitations to ask for relevant work adjustments. The result might also be affected by the fact that, at least in Sweden, SA spells due to mental diagnoses can last for a substantially longer time compared to SA due to most other conditions [35], which in itself might be a hinder for returning to work [42]. Thus, future studies should include also information on SA duration.

SA due to somatic diagnoses when aged 60–64 was also associated with not being in paid work, however, this association was less strong than that for SA due to mental diagnoses. In Sweden, like in many other countries, mental and musculoskeletal diagnoses are the most common reason for SA [43]. Among the musculoskeletal diagnoses, low back and neck pain are especially common [44], which might be a result of physically straining work conditions. Physically straining conditions, in turn, might hinder older people with somatic diagnoses to be in paid work, which could possibly be one reason for the decrease in work participation after 65. This hypothesis is supported by the result that people with lower education, who are more likely to work under physically straining conditions [45, 46], were less likely to be in paid work after 65.

At the same time, the absence of somatic SA could indicate a comparatively good work capacity among those in the older working force. Having a good work capacity might possibly imply a relatively good health status among these individuals, which in turn has been seen to predict working beyond the standard retirement age [23]. Still, it is important to note that absence of SA does not equal absence of morbidity [34]. Most morbidity does not affect people’s work capacity to the extent that they require SA or DP. Altogether, the results from this study indicate that somatic SA in the years leading up to the standard retirement age is associated with a lower likelihood of paid work after 65, although the association is perhaps not especially strong. These results are in line with prior research using other, broader measures of morbidity.

Surprisingly, prior SA due to both somatic and mental diagnoses did not show a significant association with being in paid work in the non-stratified analyses nor among women, whereas it was associated with a lower likelihood among men. Notably, the number of men was almost a one-third of the number of women within the group of SA due to both somatic and mental diagnoses. This surprising finding requires further research to explain. Future research could investigate to what degree the people who had previous SA in both somatic and mental diagnoses remained in paid work, whether they are in paid work for a shorter duration of time or work part-time to a greater extent, etc. Future research could also examine the duration and timing of the respective SA spells, and examine this in relation to the morbidities that underlie the SA spells.

### Disability pension

Unsurprisingly, prior DP was a risk factor for not being in paid work after 65 among both women and men, irrespective of diagnosis group. Nevertheless, nearly 2000 individuals with DP when aged 60–64 were in paid work after 65, i.e., approximately 25% of those with prior DP. This can seem like a very high proportion, however, it must be seen in the light of that partial DP is possible in Sweden, unlike in many other countries. Thus, some of these individuals might have been on DP for, e.g., 25% of ordinary work hours, and in part-time work the rest of the time.

Especially at older ages, when different types of morbidity are more common, work adaptations can be crucial to facilitate a prolonged working life. A recent Swedish governmental inquiry on how to promote extended working lives suggested flexible work hours, reduced work hours, and the possibility to adapt work tasks based on one’s capacity [47]. However, for those who have previously been granted DP, possibilities for work adjustments have already been assessed, and been judged as not possible.

### Strengths and limitations

Strengths of this study are the prospective cohort study design, that microdata, linked at individual level from several high-quality administrative registers [48–50] could be used for all the people in a country who fulfilled the inclusion criteria (i.e., not a sample), the large cohort allowing for sub-group analyses, that data were not self-reported (i.e., not hampered by recall bias), that there was no non-response or drop-outs, and the long study period (12 years). Another strength is that we did not include shorter SA spells (≤14 days), which means that we only include those SA spells that led to a substantial reduction of work capacity for at least more than two weeks. Consequently, shorter, often self-certified SA spells due to, e.g., the common cold, stomach flu, or a sprained ankle were not included. Also, the SA and DP diagnoses had been assessed by the patient’s treating physician and approved by the Social Insurance Agency. Additionally, we included income from work irrespective of type of employment. This also includes self-employment, which is important as many who work beyond 65 are self-employed [18].

Like every study, this study has some limitations that should be taken into consideration. We had information on income from paid work, however, not on hours worked nor on type of work. There are multiple definitions of being in paid work in the literature, based on e.g., self-reported employment status, hours worked, income, registered employment [51–53]. Our definition was based on having had registered income from work, in order to include most individuals with some degree of paid work, as many of those who work past age 65 work part-time [18, 54]. Consequently, this study’s definition of paid work was very inclusive, which could both pose as a strength and a limitation. It does mean that several heterogeneous phenomena are captured using this outcome measure. In addition, future studies might want to differentiate analyses by levels of income or number of years with earnings when aged 66–71, and differentiate between those who remain continuously in work and those who stop working and then return.

By also including those who had income from paid work at any point when aged 60–64, we avoided the health selection that can arise from excluding those who leave work via full or part-time old-age pension before the standard retirement age of 65. Also, many continue in paid work after having taken early old-age pension and can then apply for SA. However, those who retired early were also less likely to have SA or DP during the exposure window, since some of them were at risk of SA and DP for a shorter time than those who were in paid work throughout the exposure period. However, they may still have had SA before the exposure window. This means that we might have underestimated the association between SA before age 65 and not working after age 65. Since DP is granted on a permanent basis until a person turns 65, this is less likely to be an issue regarding DP.

In addition, it should be noted that the Nagelkerke R Squares were quite low (see Tables 3 and 4), indicating that besides prior SA, DP, and the sociodemographic covariates, there were other factors that can explain work participation past the age of 65 than those accounted for in this model. Such factors could, e.g., be influence by significant others [15, 55], the employer [11], societal and group norms regarding retirement [56], work environment [22, 51], motivation to continue in paid work [22], personality [57], type of morbidity, as well as duration, rate (part- or full-time), and number of SA and DP spells. Moreover, one limitation is the possible overlap between SA and DP, which might affect the results of the analysis in which SA and DP were separate exposure variables. This is also the reason why we conducted analyses with a combined variable of SA and DP.

Further, the measures of SA and DP are also relatively crude, by bundling diagnoses together into mental respectively somatic diagnoses, and not distinguishing between part-time and full-time SA and DP or having SA or DP early or late in the study period. As a result, as with work, heterogeneous phenomena may be included in each of the groups, which may lead to an over- or underestimation of the association between SA/DP and paid work after age 65. Future studies should investigate if results vary with more specific SA and DP diagnoses, with SA/DP timing, length, and rate, as well as with e.g., specific morbidities and in specific occupations.

Still, since all individuals in Sweden fulfilling the inclusion criteria were included in this study, the results can be assumed to at least be generalizable to countries with a similar public SA, DP, and old-age pension system.

## Conclusions

This study showed that previous SA due to mental diagnoses was negatively associated with being in paid work after the age of 65; the same was the case for previous SA due to somatic diagnoses, although that association was less strong. The likelihood of being in paid work was the lowest among individuals with prior DP, regardless of diagnosis group and sex. The findings fill a clear knowledge gap and indicate that mental disorders among older workers are an important aspect to consider when striving to extend people’s working lives. More knowledge of factors that affect the likelihood of being in paid work provide opportunities to target important diagnosis groups, further increasing participation in paid work in the growing group of older persons.

## Data Availability

The used data cannot be made publicly available due to privacy regulations. According to the General Data Protection Regulation, the Swedish law SFS 2018:218, the Swedish Data Protection Act, the Swedish Ethical Review Act, and the Public Access to Information and Secrecy Act, these types of sensitive data can only be made available for specific purposes that meets the criteria for access to this type of sensitive and confidential data as determined by a legal review. Professor Kristina Alexanderson (Kristina.alexanderson@ki.se) can be contacted regarding the data.

## Abbreviations

CI: Confidence interval
DP: Disability pension
ICD-10: International Classifications of Diseases 10th Revision
LISA: Longitudinal Database for Health Insurance and Labor Market Studies Registers
MiDAS: MicroData for Analysis of Social Insurance database
OR: Odds ratio
SA: Sickness absence/sick leave

## Footnotes

1 In accordance with the exchange rate on 1 January of the corresponding year.

2 In accordance with the exchange rate on 1 January of the corresponding year.

## References

[1] OECD. Aging and employment policies: Statistics on average effective age of retirement. 2013 [updated 24 jan 2020]. Available from: https://www.oecd.org/els/emp/average-effective-age-of-retirement.htm.

[2] OECD (2019). Pensions at a glance 2019.

[3] Grundy EM, Murphy M. Population ageing in Europe. In: Michel J-P, Beattie BL, Martin FC, Walston J, editors. Oxford Textbook of Geriatric Medicine. 3rd ed: Oxford University Press; 2017. p. 11–8.

[4] Lutz W, Sanderson W, Scherbov S (2008). The coming acceleration of global population ageing. Nature..

[5] OECD. Life expectancy. In: OECD, editor. Pensions at a Glance 2019: OECD and G20 Indicators. Paris: OECD Publishing; 2019. p. 172–173.

[6] Healthy life years at birth by sex [database on the Internet]. 2020. Available from: https://ec.europa.eu/eurostat/databrowser/view/tps00150/default/table?lang=en.

[7] Zack MM, Moriarty DG, Stroup DF, Ford ES, Mokdad AH (2004). Worsening trends in adult health-related quality of life and self-rated health: United States, 1993-2001. Public Health Rep.

[8] Johansson S-E, Midlöv P, Sundquist J, Sundquist K, Calling S (2015). Longitudinal trends in good self-rated health: effects of age and birth cohort in a 25-year follow-up study in Sweden. Int J Public Health.

[9] OECD. Effective age of labour market exit. In: OECD, editor. Pensions at a Glance 2019: OECD and G20 Indicators. Paris: OECD Publishing; 2019. p. 178–179.

[10] Farrants K, Marklund S, Kjeldgård L, Head J, Alexanderson K (2018). Sick leave among people in paid work after age 65: a Swedish population-based study covering 1995, 2000, 2005 and 2010. Scand J Public Health..

[11] Nilsson K (2016). Conceptualisation of ageing in relation to factors of importance for extending working life – a review. Scand J Public Health..

[12] Majeed T, Forder PM, Tavener M, Vo K, Byles J (2017). Work after age 65: a prospective study of Australian men and women. Aust J Ageing.

[13] ten Have M, van Dorsselaer S, de Graaf R (2015). Associations of work and health-related characteristics with intention to continue working after the age of 65 years. Eur J Pub Health.

[14] Eismann M, Henkens K, Kalmijn M (2019). Why singles prefer to retire later. Res Aging.

[15] Meng A, Sundstrup E, Andersen LL (2020). Factors contributing to retirement decisions in Denmark: comparing employees who expect to retire before, at, and after the state pension age. Int J Environ Res Public Health.

[16] van Solinge H, Henkens K (2014). Work-related factors as predictors in the retirement decision-making process of older workers in the Netherlands. Ageing Soc.

[17] Farrants K, Kjeldgård L, Marklund S, Head J, Alexanderson K (2017). Sick leave before and after the age of 65 years among those in paid work in Sweden in 2000 or 2005: a register-based cohort study. J Int Med Res.

[18] Wahrendorf M, Akinwale B, Landy R, Matthews K, Blane D (2017). Who in Europe works beyond the state pension age and under which conditions? Results from SHARE. Journal of Population Ageing.

[19] Virtanen M, Oksanen T, Batty GD, Ala-Mursula L, Salo P, Elovainio M (2014). Extending employment beyond the pensionable age: a cohort study of the influence of chronic diseases, health risk factors, and working conditions. PLoS One.

[20] Doshi JA, Cen L, Polsky D (2008). Depression and retirement in late middle-aged U.S. workers. Health Serv Res.

[21] Olesen SC, Butterworth P, Rodgers B (2012). Is poor mental health a risk factor for retirement? Findings from a longitudinal population survey. Soc Psychiatry Psychiatr Epidemiol.

[22] de Wind A, van der Pas S, Blatter BM, van der Beek AJ (2016). A life course perspective on working beyond retirement - results from a longitudinal study in the Netherlands. BMC Public Health.

[23] de Wind A, Scharn M, Geuskens GA, van der Beek AJ, Boot CRL (2018). Predictors of working beyond retirement in older workers with and without a chronic disease - results from data linkage of Dutch questionnaire and registry data. BMC Public Health.

[24] World Health Organization (WHO). World report on ageing and health. 2015. Available from: https://apps.who.int/iris/handle/10665/186463.

[25] Robroek S, Schuring M, Croezen S, Stattin M, Burdorf A. Poor health, unhealthy behaviors, and unfavorable work characteristics influence pathways of exit from paid employment among older workers in Europe: a four year follow-up study. Scandinavian Journal of Work, Environment & Health. 2013;39(2):125–133. 10.5271/sjweh.3319 22949091

[26] van Rijn RM, Robroek SJ, Brouwer S, Burdorf A (2014). Influence of poor health on exit from paid employment: a systematic review. Occup Environ Med.

[27] Nilsson K (2020). A sustainable working life for all ages – the swAge-model. Appl Ergon.

[28] Bird C, Rieker P, Bird C, Rieker P (2008). Gender Differences in Health. Gender and health: the effects of constrained choices and social policies.

[29] Mastekaasa A (2014). The gender gap in sickness absence: long-term trends in eight European countries. Eur J Pub Health.

[30] The National Social Insurance Board (RFV). Women, Men and Sickness Absence. Stockholm: The National Social Insurance Board (RFV), 2004. Available from: https://www.forsakringskassan.se/wps/wcm/connect/af2fbe24-98ba-41d4-b054-b0ca24022957/socialforsakringsboken_2004_eng.pdf?MOD=AJPERES.

[31] Haukenes I, Gjesdal S, Rortveit G, Riise T, Maeland JG (2012). Women's higher likelihood of disability pension: the role of health, family and work. A 5-7 years follow-up of the Hordaland Health Study. BMC Public Health.

[32] Karlsson N, Borg K, Carstensen J, Hensing G, Alexanderson K (2006). Risk of disability pension in relation to gender and age in a Swedish county: a 12-year population based, prospective cohort study. Work..

[33] Albertsen K, Lund T, Christensen K, Kristensen T, Villadsen E (2007). Predictors of disability pension over a 10-year period for men and women. Scand J Public Health.

[34] Wikman A, Marklund S, Alexanderson K (2005). Illness, disease, and sickness absence: an empirical test of differences between concepts of ill health. J Epidemiol Community Health.

[35] Lidwall U (2015). Sick leave diagnoses and return to work: a Swedish register study. Disabil Rehabil.

[36] Hensing G, Spak F (1998). Psychiatric disorders as a factor in sick-leave due to other diagnoses. A general population-based study. Br J Psychiatry.

[37] World Health Organization (WHO). International statistical classification of diseases and related health problems, tenth version (ICD-10).2010.

[38] The Swedish Social Insurance Agency. Social Insurance in Figures 2011. 2011. Available from: https://www.forsakringskassan.se/wps/wcm/connect/8cbe576f-5bec-4918-863e-4bba39362d8a/socialforsakringen_i_siffror_eng_2011.pdf?MOD=AJPERES.

[39] European Commission. Your social security rights in Sweden. 2020. Available from: https://ec.europa.eu/employment_social/empl_portal/SSRinEU/Your%20social%20security%20rights%20in%20Sweden_en.pdf.

[40] The Government Offices of Sweden. The Swedish pension system and pension projections until 2070. 2018. Available from: https://ec.europa.eu/info/sites/default/files/economy-finance/final_country_fiche_se.pdf.

[41] Krupa T, Kirsh B, Cockburn L, Gewurtz R (2009). Understanding the stigma of mental illness in employment. Work..

[42] Hultin H, Lindholm C, Möller J (2012). Is there an association between long-term sick leave and disability pension and unemployment beyond the effect of health status? – a cohort study. PLoS One.

[43] The Swedish Social Insurance Agency. Social Insurance in Figures 2020. 2020. Available from: https://www.forsakringskassan.se/wps/wcm/connect/cf56c741-1668-43c2-887d-10dd6c243cde/social-insurance-in-figures-2020.pdf?MOD=AJPERES&CVID=.

[44] The Swedish Council on Technology Assessment in Health Care (SBU). Back and Neck Pain: An Evidence Based Review. Summary and Conclusions. Stockholm: The Swedish Council on Technology Assessment in Health Care (SBU), 2000 145. Available from: https://www.sbu.se/contentassets/a1c7fd6945514079bf0db408269e6685/backpainslut.pdf.

[45] Sainio P, Martelin T, Koskinen S, Heliövaara M (2007). Educational differences in mobility: the contribution of physical workload, obesity, smoking and chronic conditions. J Epidemiol Community Health.

[46] Kjellberg K, Lundin A, Falkstedt D, Allebeck P, Hemmingsson T (2016). Long-term physical workload in middle age and disability pension in men and women: a follow-up study of Swedish cohorts. Int Arch Occup Environ Health.

[47] The Swedish Government Official Reports. Äldre har aldrig varit yngre – allt fler kan och vill arbeta längre. [Older people have never been younger - more and more people can and want to work longer]. (In Swedish). SOU 2020:69. 2020. Available from: http://www.sou.gov.se/wp-content/uploads/2020/11/SOU-2020_69_webbfil.pdf.

[48] Ludvigsson JF, Almqvist C, Edstedt Bonamy A-K, Ljung R, Michaëlsson K, Neovius M (2016). Registers of the Swedish total population and their use in medical research. Eur J Epidemiol.

[49] Ludvigsson JF, Svedberg P, Olén O, Bruze G, Neovius M (2019). The longitudinal integrated database for health insurance and labour market studies (LISA) and its use in medical research. Eur J Epidemiol.

[50] Ludvigsson JF, Otterblad-Olausson P, Pettersson B, U., Ekbom A. The Swedish personal identity number: possibilities and pitfalls in healthcare and medical research. Eur J Epidemiol 2009;24(11):659–667. 10.1007/s10654-009-9350-y PMC2773709 19504049

[51] van der Zwaan GL, Oude Hengel KM, Sewdas R, de Wind A, Steenbeek R, van der Beek AJ (2019). The role of personal characteristics, work environment and context in working beyond retirement: a mixed-methods study. Int Arch Occup Environ Health.

[52] McDonough P, Worts D, Corna LM, McMunn A, Sacker A (2017). Later-life employment trajectories and health. Adv Life Course Res.

[53] Eyjólfsdóttir HS, Baumann I, Agahi N, Fritzell J, Lennartsson C (2019). Prolongation of working life and its effect on mortality and health in older adults: propensity score matching. Soc Sci Med.

[54] Lain D, Vickerstaff S. Working Beyond Retirement Age: Lessons for Policy. In: Harper S, Hamblin K, editors. International Handbook on Ageing and Public Policy: Edward Elgar Publishing; 2014. p. 242–255.

[55] Nilsson K, Rignell Hydbom A, Rylander L (2011). Factors influencing the decision to extend working life or retire. Scand J Work Environ Health.

[56] Radl J (2014). Retirement timing and social stratification a comparative study of labor market exit and age norms in Western Europe.

[57] Anxo D, Ericson T, Herbert A (2019). Beyond retirement: who stays at work after the standard age of retirement?. Int J Manpow.

